# Sarcomatoid Carcinoma of Male Urethra with Bone and Lung Metastases Presenting as Urethral Stricture

**DOI:** 10.1155/2013/931893

**Published:** 2013-10-20

**Authors:** Niraj Badhiwala, Robert Chan, Hai-Jun Zhou, Steven Shen, Michael Coburn

**Affiliations:** ^1^Washington University School of Medicine, St. Louis, MO 63110, USA; ^2^Department of Urology, Houston Methodist Hospital, Houston, TX 77030, USA; ^3^Department of Pathology, Houston Methodist Hospital, Houston, TX 77030, USA; ^4^Scott Department of Urology, Baylor College of Medicine, 6620 Main Street, Houston, TX 77030, USA

## Abstract

A 57-year-old man who presented with urinary retention was found to have a sarcomatoid carcinoma of the urethra. Evaluation with CT scan of the abdomen and pelvis revealed multiple pulmonary nodules and osteolytic lesions of left posterior ribs. After external beam radiation therapy and six cycles of systemic chemotherapy, patient underwent a surgical resection of the urethral cancer. After his surgery, patient was also found to have multiple brain metastases and underwent whole brain radiation therapy, nine months after his initial diagnosis. Sarcomatoid carcinomas of the genitourinary tract are extremely rare tumors that require a very aggressive, multimodal treatment approach.

## 1. Introduction

Sarcomatoid carcinoma (also known as carcinosarcoma) is an aggressive tumor that is very rarely seen in urinary tract. We present a case of urethral cancer that initially presented as a urethral stricture failing endoscopic management. To our knowledge, this is the first case of sarcomatoid carcinoma of male urethra without a history of prior urinary tract cancer. We also discuss the pathological study and management of urethral cancer with a literature review. 

## 2. Case Presentation

A 57-year-old man was referred with a two-year history of dysuria, weak stream, hematuria, and urinary retention. He had previously underwent two direct visual internal urethrotomy (DVIU) procedures with self-catheterization using a 14Fr catheter as well as dilation in the office. Patient was also complaining of chronic left shoulder and low back pain. On physical examination, he was noted to have urethral induration and a mass in the deep penoscrotal region and bilateral inguinal lymphadenopathy. His flow rate was 8 mL/sec with a 465 mL postvoid residual.

**Figure 1:**
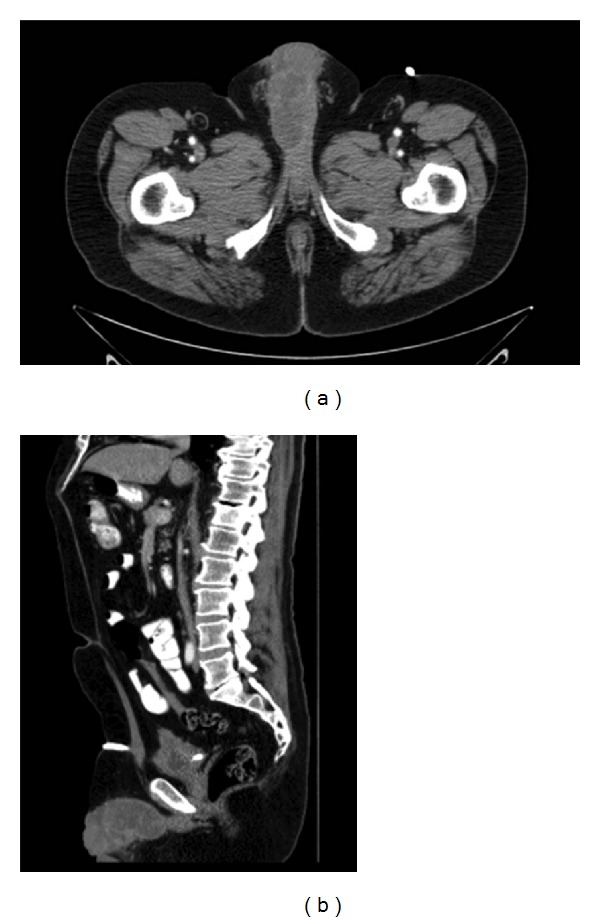
CT scan of abdomen and Pelvis with contrast showing the primary tumor. (a) Transverse view; (b) sagittal view.

CT of the abdomen and pelvis showed 5 cm heterogenous enhancing hypodense mass arising from the right side of penis that was worrisome for primary neoplasm as well as a T12 vertebral fracture. CT of the chest showed multiple pulmonary nodules and multiple osteolytic lesions in left posterior 6th and 7th ribs.  Figure 1 demonstrates CT scan of his pelvis. 

He was taken to the OR for cystoscopy, urethral biopsy of this mass, and placement of suprapubic tube. Biopsies of the urethral lesions were consistent with sarcomatoid carcinoma with no squamous features. Immunohistochemical stains were positive for pancytokeratin, CAM 5.2, and AE1/AE3, focally positive for smooth muscle actin, p63, and p53, and negative for ALK-1, CK 5/6, and CD31.

He was treated with a course of external beam radiation therapy to his left shoulder and T12 vertebra that helped his chronic pain. He underwent two cycles of cisplatin and gemcitabine based systemic chemotherapy; however, his disease continued to progress. He was switched to MVAC chemotherapy (methotrexate, vinblastine, doxorubicin, and cisplatin). After 4 cycles of MVAC chemotherapy, he underwent resection of urethral cancer mass with partial penectomy, partial scrotectomy, and total urethrectomy involving the membranous junction. There was noted to be invasion of the corpus spongiosum, corpus cavernosum, base of the penis, and perineal soft tissue. On postoperative day 2, he developed new-onset seizure-like activity involving his right arm. CT of the head demonstrated multiple hemorrhagic brain metastases. He underwent a course of whole brain radiation therapy. 

**Figure 2:**
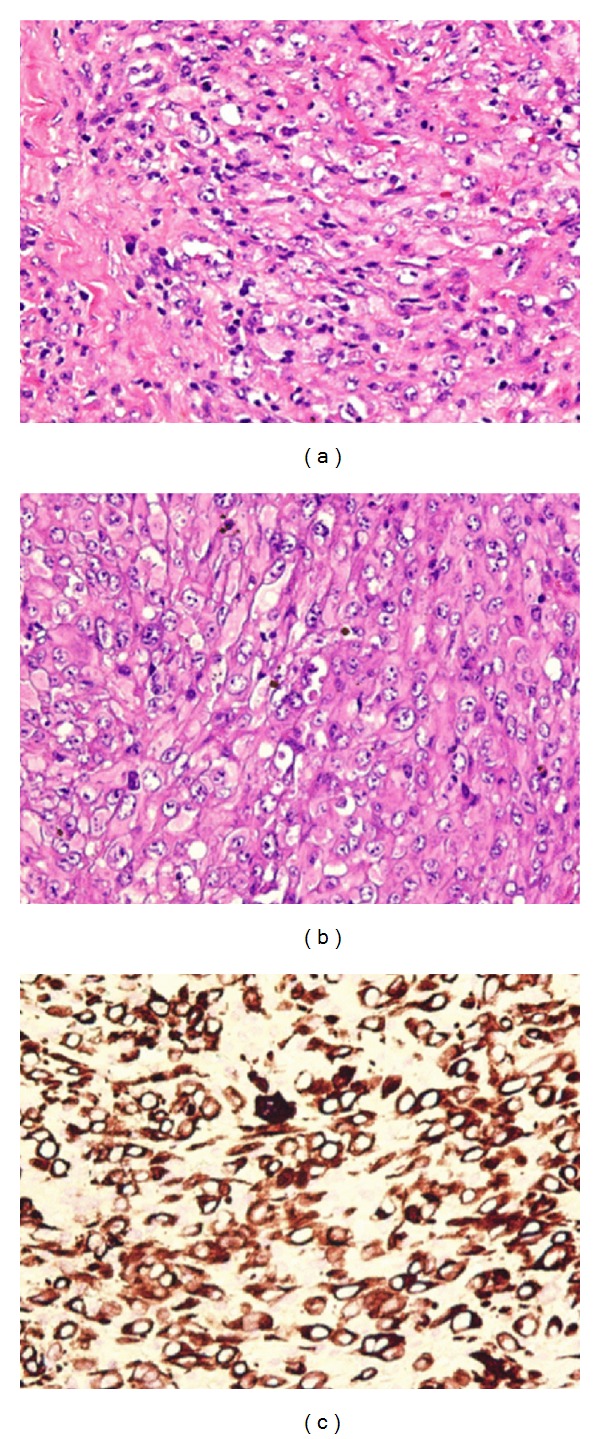
Histopathological features of this sarcomatoid urothelial carcinoma. (a) Sarcomatoid changes with areas of necrosis/hemorrhage (H&E, 200x). (b) More carcinomatoid features of the tumor cells (H&E, 200x). (c) Cytokeratin immunohistochemistry shows strong positivity of the tumor cells (200x), indicating carcinoma origin instead of true sarcoma.

The urethral mass measured 8.5 × 7.2 × 6.4 cm with high grade sarcomatoid carcinoma histology. The tumor invaded into the periurethral corpus spongiosum, the corpus cavernosum, base of penis, and the perineal soft tissue with negative margins. Final pathological staging was Stage IV. TNM staging was pT3 Nx cM1.  Figure 2 demonstrates H&E staining and cytokeratin staining at 200x magnification. 

## 3. Discussion

Sarcomatoid carcinoma is an aggressive tumor that can occur in any organ. These tumors are composed of both malignant mesenchymal and epithelial elements. It is very rarely seen in the urinary tract, and only a very limited number of cases in the renal pelvis and ureter [1–5], bladder [6–9], and female urethra [10–14] have been documented. Only one case of sarcomatoid carcinoma has been reported in a male urethra with a history of radical cystectomy for transitional cell carcinoma. To our knowledge, this is the first case of sarcomatoid carcinoma of male urethra without a history of prior urinary tract cancer.

This patient initially presented with recurrent urethral stricture that failed multiple endoscopic treatments. Since urethral neoplasms are rarely seen and urethral strictures occur quite frequently, diagnosis of urethral cancer is usually delayed [15]. The possibility of urethral cancer should be considered whenever there is progressive difficulty in controlling a stricture. 

These tumors are usually very aggressive possibly because they are diagnosed at an advanced stage [16]. A complete workup including a thorough history and physical exam as well as imaging studies to evaluate for metastasis should be performed. Transurethral biopsy to confirm the diagnosis is an essential component of the workup. 

Although sarcomatoid carcinoma in the urethra is a rare event, the prognosis in these cases appears to be very poor.  Table 1 reviews the 4 reported cases of urethral sarcomatoid carcinoma. Three out of the four reported cases had metastatic disease present at the time of presentation. Mean followup was 14 months, and one patient died within 5 months of presentation. Dalbagni et al. evaluated 46 men with primary carcinoma of male urethra and reported overall survival at 5 years 42% for all tumors and 36% for invasive tumors [17]. Radiation therapy or surgery alone has been ineffective in the management of male urethral carcinoma [18, 19]. Multimodal therapy with chemoradiation and surgical resection of the tumor has produced better results [17]. This aggressive disease at an advanced stage warrants collaboration between the medical oncologist, the radiation oncologist, and the urologist.

## 4. Conclusion

Since this rare but aggressive cancer to male urethra mimics urethral stricture, every effort should be made to establish an early diagnosis and aggressively treat them using a multimodal approach.

## Figures and Tables

**Table 1:** Sarcomatoid carcinoma in the urethra.

Authors	Patient	Symptoms and clinical findings	Location, size, and histology of tumor	Outcome
Liu & Wu [11] 2011	50 y.o. female	Urinary tract infection & urinary retention	3 × 1 × 1 cm tumor that was high grade papillary serous carcinoma with psammoma bodies & mesenchymal component with area of heterologous (cartilaginous) element; epithelioid tumor cells containing melanin pigment	Total cystourethrectomy, total vaginectomy, bilateral PLND, and ileal conduit urinary diversion were performed

Komai et al. [10] 2005	78 y.o. female	Bleeding in the region of the genitalia	Sarcomatoid carcinoma composed of SCC and spindle cell sarcoma	Urethrectomy & suprapubic cystostomy; radiation therapy of 50 Gy to recurrence site; alive at 26 mo f/u

Konno et al. [13] 1997	61 y.o. female	Acute urinary retention	4 × 5 × 5 cm tumor that was carcinosarcoma with heterologous differentiation with heterologous differentiation in the region of the urethra, including a carcinoma, adenocarcinoma, and partly SCC and chondrosarcoma.	Tumor excision using transperineal approach and urinary diversion by cystostomy; local radiation 50 Gy; at 12 month f/u tumor metastasis to the pelvic bone

Xu et al. [14] 1993	83 y.o. male	15 years after an RC for TCC, with intermittent painless bloody discharge and presence of papillary mass at external ureteral meatus	Tumor that arose from the penile urethra and invaded the glans penis and penile corpus cavernosum; tumor composed of mixed carcinoma (SCC, adenocarcinoma, TCC) and rhabdomyosarcoma	Total phallectomy, scrotectomy, orchiectomy, and urethrectomy; metastasis to right inguinal node at the time of presentation; patient died after 5 months

CT: computed tomography; SCC: squamous cell carcinoma; TCC: transitional cell carcinoma; RC: radical cystectomy.
